# A rare association between factor H deficiency and lupus: Case report and experimental treatment with curcumin

**DOI:** 10.3389/fped.2022.1039291

**Published:** 2022-11-04

**Authors:** Ana Catarina Lunz Macedo, Lazara Elena Santisteban Lores, José Antonio Tavares Albuquerque, Nilo José Coelho Duarte, Paschoalina Romano, Persio Almeida Rezende Ebner, Vinicius Marcondes Rezende, Clovis A. Silva, Luís Eduardo Coelho Andrade, Dewton Moraes Vasconcelos, Lourdes Isaac

**Affiliations:** ^1^ Pediatric Nephrology Unit, Hospital das Clinicas HCFMUSP, Faculdade de Medicina, Universidade de Sao Paulo, São Paulo, Brazil; ^2^ Department of Immunology, Institute of Biomedical Sciences, Universidade de São Paulo, São Paulo, Brazil; ^3^ Laboratory of Medical Investigation – LIM 03- Central Laboratory Division, Hospital das Clinicas HCFMUSP, Faculdade de Medicina, Universidade de São Paulo, São Paulo, Brazil; ^4^ Pediatric Rheumatology Unit, Hospital das Clínicas HCFMUSP, Faculdade de Medicina, Universidade de São Paulo, São Paulo, Brazil; ^5^ Rheumatology Division, Escola Paulista de Medicina, UNIFESP, São Paulo, Brazil; ^6^ Laboratory of Medical Investigation in Dermatology and Immunodeficiencies – LIM 56, Institto de Medicina Tropical, Hospital das Clinicas HCFMUSP, Faculdade de Medicina, Universidade de São Paulo, São Paulo, Brazil

**Keywords:** FH deficiency, lupus, curcumin, experimental treatment, translational medicine, case report

## Abstract

Factor H (FH) is one of the most important regulatory proteins of the alternative pathway of the complement system. FH deficiency is a rare condition that causes unregulated C3 consumption, leading to an increased susceptibility to infections and glomerulopathies. Our previous studies have demonstrated a FH deficient patient carrying a c.452G > A, *p*.R127H FH mutation which leads to a misfolded protein and its retention in the endoplasmic reticulum. In his cultured fibroblasts, FH-delayed secretion was partially rescued when treated with curcumin, and once secreted, exhibited normal regulatory function. Here, we report a childhood-onset systemic lupus erythematosus (cSLE) in this FH deficient patient and the results of experimental treatment with curcumin aiming to rescue FH secretion and regulatory activity.

## Introduction

Factor H (FH) is a central regulator of the complement system that inhibits excessive alternative pathway activation by acting as a cofactor for Factor I in the cleavage of C3b into iC3b. FH also competes with Factor B (FB) for binding to C3b, resulting in the dissociation of Bb from C3- and C5-convertases. FH deficiency is related to C3 deficiency owing to the acceleration of C3 consumption. Complete homozygous FH deficiency is a rare condition, and to date a few cases have been described (1, 2). In humans, a single gene on chromosome 1q32 codes for FH. Mutations in the human *FH* gene can result in the lack of protein in the plasma due to misfolding and impaired protein secretion or in normal range detection but with defective regulatory functions (3). FH deficiency is mainly associated with an increased susceptibility to infections, and patient's defective regulatory functions can lead to C3-glomerulopathy, atypical haemolytic uraemic syndrome, and age-related macular degeneration (1–5). FH- and C3-deficient patients are treated constantly with antibiotics and when in critical conditions, they are plasma infused to replace complement proteins levels. Clearly, new options of treatment are required. In experimental models, the use of mini-FH (containing domains 1–4 and 19–20) offer promising results in the regulation of the alternative pathway (6). However, so far this has not been tested in FH-deficient patients.

We have previously described the case of a patient with FH deficiency diagnosed after two episodes of complicated pneumonia (7). The homozygous variant c.452G > A in CFH determining *p*.R127H was found in the patient by Sanger sequencing analysis (7). *In vitro*, the patient's fibroblasts retain FH in the endoplasmic reticulum, resulting in delayed secretion. These cells, when treated with curcumin, showed increased secretion of the mutant FH, which once transported, had normal regulatory function in the alternative pathway of the complement system (8).

Curcumin is a hydrophobic polyphenol derived from the rhizome of the *Curcuma longa* plant with a broad spectrum of antioxidant, anti-inflammatory, antimicrobial, and anticancer effects. It is well accepted that curcumin has low bioavailability in humans, chemical instability, and rapid metabolism, even when administered orally at doses as high as 12 g/day (9). A formulation of curcumin in nanoparticles (Theracurmin®) has been used in clinical trials, presenting better absorption and bioavailability (10, 11). Based on these observations, we hypothesised that Theracurmin® could be used to treat FH deficiency *in vivo*.

Before the experimental treatment, the patient presented unexpected signs and symptoms of childhood-onset systemic lupus erythematosus (cSLE). SLE is a rare autoimmune disorder that affects multiple organs and systems (12–14). Deficiencies in the first components of the classical pathway C1q/r/s, C4, and C2 are frequently associated with early onset SLE or lupus-like disease (15). However, deficiency of alternative pathway proteins has rarely been associated with SLE development (1).

In this case report, we discuss the diagnosis of cSLE in this patient and the results of treatment with Theracurmin® for his FH deficiency.

## Case description

A 2-year-old Brazilian boy, the second child of first-degree cousins with Japanese ascendance, was hospitalised in the intensive care unit because of complicated pneumonia with bilateral pleural effusion. The patient was treated with antibiotic therapy and bilateral thoracic drainage with complete resolution. At the age of 3 years, he presented with a new episode of pneumonia that required invasive ventilation and antibiotic therapy, and was discharged after three weeks. He was fully vaccinated according to the public health schedule available when he was a child, which at that time did not include pneumococcal vaccine. After hospitalisation, prophylactic amoxicillin was prescribed. Immunological evaluation revealed FH deficiency, associated with reduced levels of C9, C3, and FB owing to the lack of regulation of the alternative pathway. The variant c.452G > A in CFH, which determines *p*.R127H, was found in the homozygous patient by Sanger sequencing analysis, and this variant was found in both parents in heterozygosis (7). He had chickenpox with no complications at 6 years of age, despite not vaccinated. At 15 years of age, he presented with a malar rash and photosensitivity, with periods of improvement and worsening. One year later, the patient developed persistent rashes on the posterior cervical and thoracic region. At the age of 17, he reported episodes of acute and painful oligoarthritis involving the right elbow, right ankle, and right fourth finger (proximal interphalangeal joint). These episodes lasted two to four days, and improved after a short course of non-steroidal anti-inflammatory drugs.

At 18 years of age, when he was invited to participate in the experimental treatment protocol, he had a malar rash, photosensitivity, and posterior cervical and thoracic rash, and had reported episodes of oligoarthritis. Ultrasound examination performed during a new episode of acute arthritis revealed normal radiographic findings and small joint effusion with synovial thickening in the olecranial fossae of the elbow. Despite the distribution of the rashes in the posterior cervical and thoracic region, the lesions were not compatible with dermatomyositis, and skin biopsies confirmed the immunofluorescence pattern characteristic of the skin lesion in SLE. Thoracic lesion skin biopsy showed lupus band test with immunofluorescence of continuous cross-linked IgM and focal granular IgG, with the absence of IgA and C3 along the dermoepidermal junction. IgG fluorescence was also observed in the keratinocyte nuclei. Ophthalmic exam did not reveal any abnormalities or pigmentary changes in the retina. The laboratory findings showed haemoglobin, 14.6 g/dl; white blood cell count, 6,560/mm³ (47% neutrophils, 32% lymphocytes, 5% eosinophils, 1% basophils, and 15% monocytes); and platelet count 300,000/mm³. C reactive protein was 2.18 mg/dl, C3 < 4 mg/dl (reference 67–149 mg/dl), C4 26,7 mg/dl (reference 10–38 mg/dl), C1q 389 mg/dl (reference 100–250 mg/dl), and C2 14,1 (reference 14–25 mg/dl). Anti-C1q 32 U/ml (reference <9 U/ml). Immunological tests showed antinuclear antibodies on HEp-2 cells (HEp-2 ANA) 1:1,280 thick speckled pattern, positive ENA (anti-Sm 42,8 U/ml, anti-RNP >200 U/ml, anti-Ro/SSA >200 U/ml, anti-La/SSB 35,92 U/ml—reference values <15 U/ml) IgG 21.58 GPL/ml anticardiolipin (reference <10 GPL/ml) and IgM anticardiolipin 26.11 MPL/ml (reference <7 MPL/ml) autoantibodies. Rheumatoid factor was positive, with 219.2 U/ml (reference value <14 U/ml). Anti-double-stranded DNA (anti-dsDNA) antibodies, ANCA (antineutrophil cytoplasmic antibodies), anti-JO1 (anti-histidyl T-RNA synthetase), and anti SCL-70 (anti-topoisomerase I) were negative. Urinalysis did not indicate the presence of leukocytes or erythrocytes and the albumin/creatinine ratio was 9.2 mg/g. The serum urea and creatinine levels were 18 mg/dl and 0.79 mg/dl, respectively.

## Treatment description

**Figure 1:**
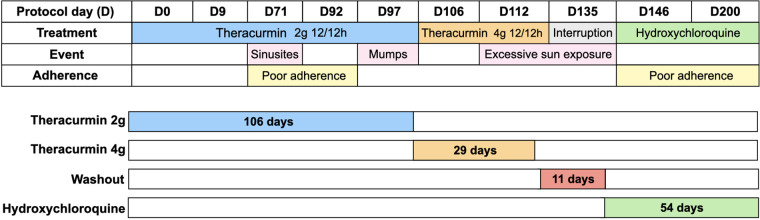
Protocol design, evaluation time, therapeutic intervention, events and report of treatment adherence.

Theracurmin® was kindly provided by Theravalues Corporation (Tokyo, Japan). After obtaining informed consent and local ethical committee approval, we initiated the regimen of a 2-g dose of Theracurmin® powder (containing 200 mg curcumin), diluted in water, orally every 12 h for 106 days. The dosage was subsequently increased to a 4-g dose of Theracurmin® every 12 h for an additional 29 days. As indicated in Figure 1, during treatment with Theracurmin®, the patient was not treated with standard treatment for SLE. He first received Theracurmin® alone and, after the washout, started standard treatment with hydroxychloroquine. During treatment, the patient was closely observed for any worsening in his condition, which would lead to an immediate discontinuation of the protocol.

Plasma levels of FH and factors B and C3 were assessed before and throughout the treatment. Plasma levels of IL-1β, IL-6, IL-8, IL-10, IL-12p70, and TNF-α were quantified before the first dose (D0), and on D9 (Table 1).

**Table 1:** Concentrations of the cytokines IL-1ß, IL-6, IL-8, IL-10, IL-12p70 and TNF-α were assessed pre-treatment at D0 and D9 in regular use of Theracurmin® 2-g every 12 h.

Cytokine	D0 (fg/ml)	D9 (fg/ml)
IL-6	297	68
IL-8	4,008	3,103
IL-10	1,002	1,183
IL-12p70	506	553
IL1-ß	1,188	1,882
TNF-α	1,091	1,377

Tolerance and side effects were assessed using clinical and laboratory parameters throughout the protocol. Lupus activity was measured using SLEDAI-2 K (16). The patient's quality of life was assessed using the Paediatric Quality of Life Inventory 4.0 - PedsQL 4.0 - (17), covering four domains: health and activities; emotional aspects; social aspects; and school performance before treatment (D0), D135, and D200. During the protocol, the patient had sinusitis, mumps despite being vaccinated, and self-limiting episodes of arthritis. Our patient reported poor compliance at two time points during the treatment. These events are illustrated in Figure 1.

## Results and discussion

### SLE and FH deficiency

To confirm the diagnosis of SLE, clinical and laboratory findings were correlated with the scoring criteria from the American College of Rheumatology (ACR) (18), Systemic Lupus International Collaborating Clinics (SLICC) (19), and the EULAR/ACR classification (EULAR) (20). Although low levels of C3 are primarily caused by FH deficiency, SLE was diagnosed in this patient by all three classifications. Since the patient developed clinical signs of lupus before the age of 18, cSLE was confirmed.

The patient presented with more than 6 of the 11 points of ACR classification. The sensitivity and specificity of the 1982 ACR criteria for paediatric lupus are 96% and 100%, respectively (21). Without considering the low level of C3, the patient met two clinical and two immunological criteria of the SLICC classification. In the EULAR/ACR classification, the patient scored more than 10 points. Although the characteristic skin biopsy is not scored as a criterion, immunofluorescence performed on biopsy reinforces lupus diagnosis.

To confirm that cSLE was absent at 3 years of age, when he was diagnosed with primary immunodeficiency, the same autoantibodies were also tested in the patient's frozen serum samples. The following auto antibodies were analysed: HEp-2 ANA, anti-DNA, ANCA, anti-JO1, anti SCL-70, RNP-SM, anti-SM, anti-Ro, anti-La, anticardiolipin IgG, and anticardiolipin IgM. All titres were negative, except for HEp-2 ANA (1:320). Autoantibodies can be present many years before the diagnosis of SLE (22), but for this patient, the retrospective assessment for lupus was negative at 3-year-old, confirming that cSLE occurred after the primary immunodeficiency.

Case reports that relate autoimmune diseases to the complement system are often related to the deficiency of components of the classical pathway and this association between immunodeficiency and autoimmune diseases has been well-documented in the literature (23). Turley et al. (2015) studied 77 complement-deficient patients and observed that 37% of those presented defects in the classical pathway had SLE-like disease (24). The most remarkable genetic association with SLE is the high frequency of deficiencies in early classical pathway components, mainly C1q (90%–93%), C1r/C1s (50%–57%), C4 (75%), and C2 (10%). These patients usually present with SLE at an early age, with severe symptoms and poor prognosis (15). However, the association between SLE and deficiencies in the components of the alternative pathway is unusual. Homozygous FH deficiency is a rare phenomenon and only a few cases have been reported (2). There are only two cases in the literature of concomitant FH deficiency and SLE (25, 26). In one case, a Caucasian daughter of non-consanguineous parents presented with subacute cutaneous lupus, with ANA and anti-DNA positivity, FH deficiency, and undetectable C3 serum levels. C2 levels were reduced, with normal serum C4 and C1q levels. Until the period when the case was published at age 59, this patient did not present with glomerulonephritis (25). In another case, a Caucasian daughter of consanguineous first-degree cousins had arthritis, fever, erythema malar, anaemia, nephritis, ANA positivity, and intense fluorescence for IgG in the nuclei of keratinocytes in the skin biopsy. The patient had undetectable serum concentrations of FH, C3, and FB; low concentrations of C2; and low levels of C1q and C4, with normal C1-INH (26). These two cases reported FH deficiency and lupus. They also had deficiencies in the initial components of the classical pathway, which could justify their association with lupus. Here, we describe a rare association of a homozygous deficiency in FH, a regulatory protein of the alternative pathway, and cSLE. To the best of our knowledge, there is no previous case of a patient who initially presented with FH deficiency at an early age, with normal components of the classical pathway, and cSLE development during adolescence. To date, we cannot speculate which pathophysiological mechanisms could explain a possible association between FH deficiency and SLE. Possibly, there are underlying genetic variants that may predispose to SLE, which we have not been able to explore until now.

### Curcumin treatment evaluation

Patient plasma concentrations of curcumin were assessed at the beginning of the treatments with 2-g and 4-g doses of Theracurmin®. Blood samples were collected immediately before and at 30 min, 60 min, 90 min, 2 h, 4 h, 6 h, 8 h, 12 h, 24 h after the administration of the first 2-g dose of Theracurmin®. The same protocol was performed with the first 4-g dose Theracurmin® (there was no washout period during treatment).

There are studies using doses as low as 500 mg up to 12 g with good tolerance and minor adverse events. The different curcumin treatment regimens and formulations available in the literature are not comparable with each other and there is no consensus on their use. We designed this specific protocol with Theracurmin® because its better absorption might be an advantage for its clinical function (27–29).

It was possible to identify through HPLC-MS/MS that the highest plasma concentration of curcumin throughout the treatment was 432 ng/ml (1,17 µM), which was reached immediately after the first dose. The plasma curcumin concentration after the very first 2-g dose Theracurmin® was consistently higher than the results obtained after chronic use, even with a 4-g dose as well as throughout the treatment. This finding of lower plasma curcumin concentrations in chronic use despite the increased dose of Theracurmin® has not been previously explained in the literature.

A previous report assessed plasma curcumin levels in healthy volunteers after a single oral 150-mg dose followed by a 210-mg dose of Theracurmin®, separated by a washout period of two weeks between dose escalation. They demonstrated an increase in plasma curcumin levels in a dose-dependent manner (10).

Oral curcumin is known to have low bioavailability owing to its low absorption by the small intestine, coupled with extensive reductive and conjugative metabolism in the liver and elimination through the gall bladder. After ingestion, curcumin is subsequently reduced in enterocytes and hepatocytes by a reductase to dihydrocurcumin, tetrahydrocurcumin, hexa-hydrocurcumin, and octahydrocurcumin, which can be found in free forms or as glucuronides (9). Several studies have been performed using oral curcumin powder supplements to investigate their absorption. Degradation reactions change the structure and properties of curcumin, thereby affecting its pharmacokinetic and pharmacodynamic behaviour (9).

**Figure 2:**
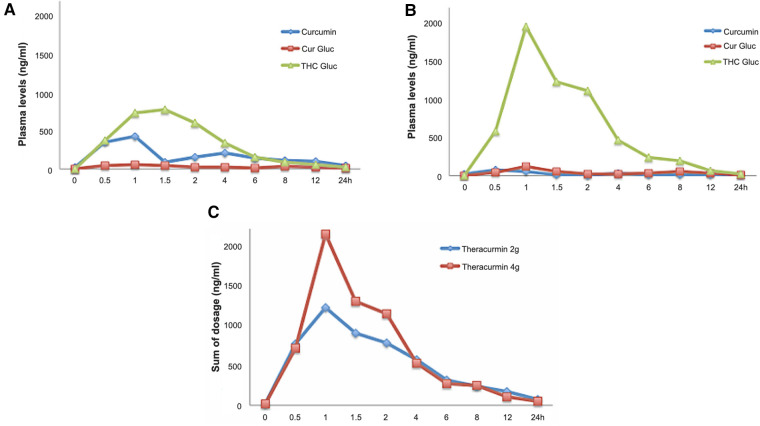
Curcumin and metabolites levels in patients's plasma. (**A**) 2-g Theracurmin®; (**B**) 4-g Theracurmin®; (**C**) Sum comparison of all curcumin fractions and metabolites with 2-g and 4-g Theracurmin®. Cur Gluc: Curcumin Glucuronidase; THC Gluc: Tetrahydrocurcumin Glucorunidase.

We speculate that our finding of lower plasma curcumin levels despite increasing (doubling) the dose of Theracurmin® in a continuous treatment could be an effect of enzymatic induction and tolerance with acceleration of the drug's metabolism over time (30–33). Both curcumin and its major reduced forms (dihydrocurcumin, tetrahydrocurcumin, and hexahydrocurcumin) can undergo glucuronidation. To clarify these questions, plasma samples were treated with glucuronidase, and the amounts of curcumin and tetrahydrocurcumin were assessed using HPLC-MS/MS for comparative analysis. This additional evaluation showed high concentrations of plasma curcumin after the first 2-g dose of Theracurmin®, whereas high levels of its metabolite, tetrahydrocurcumin, were observed after continuous use, assessed with 4-g dose of Theracurmin® (Figure 2).

Albuquerque et al. (2012) used 2 µM pure curcumin (Sigma-Aldrich) in the patient's fibroblast culture (8). The *in vitro* concentration of curcumin in that experiment was high, and this was not achievable in the *in vivo* treatment protocol because curcumin is unstable under physiological conditions and rapidly metabolised to other products.

Plasma levels of FH, FB and C3 were assessed before and throughout the treatment. Despite the levels of curcumin and its metabolites in the patient's plasma, the treatment did not affect the concentration of FH, FB and C3.

The plasma concentrations of IL-1β, IL-6, IL-8, IL-10, IL-12p70, and TNF-α were determined before treatment (D0) and on D9 under regular use of Theracurmin® 2 g every 12 h (Table 1). Considering the cytokine response to treatment, there was a marked drop in the plasma concentration of IL-6, and a reduction of approximately 20% in IL-8 levels. However, IL-1β increased more than 50%, and slight increases in TNF-α, IL-10, and IL-12p70 levels were observed. A meta-analysis of nine randomised controlled studies demonstrated that IL-6 levels were reduced by treatment with curcumin, and this effect was more evident in more intense inflammatory states. Our results only partially agree with the literature, which describes the reduction in serum concentrations of IL-1β, IL-6, IL-8, IL-12, and TNF-α with the use of curcumin (34).

To ensure the safety of the treatment, the patient was closely monitored and lupus activity was measured using SLEDAI-2K throughout the treatment. SLEDAI-2K remained stable, as did haematologic, renal, and hepatic parameters (Table 2). Since the low level of C3 is secondary to FH deficiency, we did not consider C3 in the SLEDAI-2K; however, even if it were considered, it would have remained at 4 (a low activity index) throughout the protocol. We concluded that neither the introduction nor interruption of curcumin treatment interfered with lupus activity.

**Table 2:** Laboratorial tests assessed pre-treatment at D0, D113 in regular use of Theracurmin® 4 g every 12 h, D147 after 11 days washout, and D201 in regular use of Hydroxychloroquine 400 mg.

Protocol day (D)	D1	D113	D147	D201
Serum creatinine (0.70–1.20 mg/dl)	0.79	0.88	0.85	0.8
Serum urea (13–43 mg/dl)	18	22	27	25
Urine density U (1003–1029)	1,020	1,020	1,015	1,020
Urine pH (4.50–7.80)	6	6	6	5
Urine leucocytes (0–7,000)	0	1,000	1,000	1,000
Urine red blood cells (0–3,000)	0	1,000	0	500
Urine protein/creatinine ratio (0.06–0.2 g/g)	0.1	0.06	NA	0.07
Urine albumin/creatinine ratio (<30 mg/g)	9.2	6.58	NA	5.69
Urine NGAL (0.40–72 ng/ml)	7.61	6.69	2.19	NA
C-reactive protein (<5 mg/dl)	2.2	1.87	2.77	1.08
White blood cell count (4,500–11,000/mm^3^)	6,560	7,100	5,030	5,480
Neutrophils (45,5–69,5%)	47	67	50	44
Lymphocytes (20,3–47%)	32	17	27	32
Monocytes (1,6–10%)	15	11	15	13
Eosinophils (0–4,4%)	5	5	7	10
Basophils (0,2–1,2%)	1	0	1	1
Haemoglobin (13.5–17.5 g/dl)	14.6	14.3	13.2	14.2
Platelet count (150–450×103/mcL)	300	374	261	318
Aspartate transaminase (15–40 U/L)	16	15	29	17
Alanine transaminase (10–40 U/L)	18	21	29	15
Bilirubin (<1 mg/dl)	0.64	0.36	0.54	0.62
Gamma-glutamyltransferase (2–30 U/L)	22	20	18	16
Cholesterol (<150 mg/dl)	133	127	119	131
Triglycerides (<100 mg/dl)	91	107	86	62
Serum total protein (6.0 a 8.0 g/dl)	7.6	7.8	7.7	8.2
Serum albumin (3.2 a 5.0 g/dl)	3.9	3.9	4.1	4.4
Serum globulin (0.7 a 1.5 g/dl)	1.7	1.9	1.7	1.8
C1q (100–250 mg/dl)	389	NA	NA	NA
C2 (14–25 mg/dl)	14.1	NA	NA	NA
C3 (67–149 mg/dl)	<4	5	<4	<4
C4 (10–38 mg/dl)	26.7	27.8	19.8	21.4
HEp-2 ANA (non-reactive)	1:1,280	NA	NA	NA
Anti-double-stranded DNA (<1.0)	NR	NA	NA	NA
Anti-C1q (<9.0 U/ml)	32	NA	NA	NA
Anti-RNP (non-reactive—U/ml)	>200	NA	NA	NA
Anti-Sm (non-reactive—U/ml)	42.8	NA	NA	NA
Anti-Ro/SSA (non-reactive—U/ml)	>200	NA	NA	NA
Anti-La/SSB (non-reactive—U/ml)	35.9	NA	NA	NA
IgG anti-cardiolipin (<10 U GPL/ml)	21.5	NA	NA	NA
IgM anti-cardiolipin (<10 U MPL/ml)	26.1	NA	NA	NA
ANCA (non-reactive)	NR	NA	NA	NA
Anti-JO1 (non-reactive)	NR	NA	NA	NA
Anti SCL-70 (non-reactive)	NR	NA	NA	NA
Rheumatoid factor (<14 U/ml)	219.2	NA	NA	NA

Normal values are indicated in parentheses. NR: non-reactive; NA: not available; NGAL: Neutrophil gelatinase-associated lipocalin; ANCA: Antineutrophil cytoplasmic antibodies; Anti-JO1: Anti-histidyl T-RNA synthetase; Anti SCL-70: Anti-topoisomerase I; HEp-2 ANA: Antinuclear antibodies on HEp-2 cells.

We conclude that the patient showed good tolerance to both doses of Theracurmin® without adverse symptoms or side effects, as reported in previous publications.

## Patient perspective: PedsQL 4.0

The perception of quality of life by PedsQL 4.0 was assessed before treatment on D0, on D135 (during treatment with Theracurmin® 4 g every 12 h) and on D200 (after discontinuation of Theracurmin® and under treatment with hydroxychloroquine 400 mg). In the PedsQL 4.0, the best quality of life was related to the lowest score. In the patient's opinion, the scores were 11, 15, and 4 at the respective three time points. Parents' views on adolescents’ quality of life were 17, 19, and 2 at the same time points. Considering the four domains, there was a significant improvement in quality of life, mainly in health and activities, in the third assessment under use of hydroxychloroquine.

## Conclusion

We have reported a rare case of FH deficiency and cSLE. Oral curcumin supplementation for FH deficiency was well tolerated with no adverse effects. Despite the previous success of *in vitro* treatment of patient fibroblasts, *in vivo* treatment did not result in an increase in the plasma levels of FH, C3, and FB. Although the plasma concentrations of IL-6 and IL-8 declined and IL-1β increased after the treatment, no improvement or burden in the clinical and laboratory cSLE parameters were observed.

It is well established that curcumin has low bioavailability and that it can be ameliorated by new formulations. In our case, using a nanoparticle formulation (Theracurmin®), the plasma levels of curcumin rapidly decreased with continuous use, whereas the level of tetrahidrocurcumin increased. These results could be due to enzymatic induction and tolerance with acceleration of the drug's metabolism over time and might justify the absence of an effect of curcumin treatment for this patient.

For this patient, personalised translational medicine showed that previous successful *in vitro* treatment could not be assumed as a beneficial long-term treatment for FH deficiency. More efforts are needed to clarify curcumin bioavailability, absorption, reductive, and conjugative metabolism during short-term and long-term treatment.

## Data Availability

The raw data supporting the conclusions of this article will be made available by the authors, without undue reservation.
